# Heart and Mind: Essential Components in Sound Decision-Making

**DOI:** 10.1093/geroni/igaa057.1171

**Published:** 2020-12-16

**Authors:** Michaela Clark, Julie Hicks Patrick, Michaela Reardon

**Affiliations:** West Virginia University, Morgantown, West Virginia, United States

## Abstract

Consumer tasks permit an ecologically-valid context in which to examine the contributions of affective and cognitive resources to decision-making processes and outcomes. Although previous work shows that cognitive factors are important when individuals make decisions (Patrick et al., 2013; Queen et al.), the role of affective components is less clear. We examine these issues in two studies. Study 1 used data from 1000+ adults to inform a cluster analysis examining affective aspects (importance, meaningfulness) of making different types of decisions. A 4-cluster solution resulted. In Study 2, we used affective cluster membership and cognitive performance as predictors of experimental decision-making outcomes among a subset of participants (N = 60). Results of the regression (F(2, 40) = 6.51, p < .01, R2 = .25.) revealed that both the affective clusters (b = .37, p = .01) and cognitive ability (b = -.30, p = .04) uniquely contributed to the variance explained in decision quality. Age did not uniquely contribute. Results are discussed in the context of developing measures that enable us to move the field forward.

